# Biopsychosocial Correlates of Adjustment to Cancer during Chemotherapy: The Key Role of Health-Related Quality of Life

**DOI:** 10.1155/2019/9750940

**Published:** 2019-03-10

**Authors:** Marco Lauriola, Manuela Tomai

**Affiliations:** ^1^Department of Social and Developmental Psychology, Sapienza University of Rome, Italy; ^2^Department of Dynamic and Clinical Psychology, Sapienza University of Rome, Italy

## Abstract

**Background:**

Patients adjust to cancer in a continuous process that follows the course of the disease. Previous research has considered several illness-related variables and demographics, quality of life, personality, and social factors as predictors of adjustment to cancer, which can be maladaptive (e.g., helplessness-hopelessness and anxious preoccupation) or adaptive (e.g., fighting spirit).

**Aims:**

Assuming a biopsychosocial view, we test an empirical model in which disease stage, patient's age, and gender are viewed as the distal antecedents of positive and negative adjustment to cancer for chemotherapy patients. Health-related quality of life (HRQoL) has a key role, interposing between the distal antecedents and adaptational outcomes. Social support and positive thinking are also included in the model as related to adjustment.

**Methods:**

One-hundred-sixty-two consecutive cancer patients receiving adjuvant or standard chemotherapy participated in the study. Patients completed the Mini-Mental Adjustment to Cancer, the Brief-COPE, the Social Provision Scale, and the SF-12 Health Survey. Partial least squares structural equation modeling (PLS-SEM) was applied for model building and hypotheses testing.

**Results:**

We found a negative association between advanced stage and physical functioning, a strong positive link between physical functioning and mental health, and significant relations between mental health and helpless-hopelessness, anxious preoccupation, and cognitive avoidance. Social support and positive thinking were related to fighting spirit and fatalism. Cancer stage and female gender were indirectly associated with adaptational outcomes through HRQoL. The patient's age had no significant relationships in the model.

**Discussion:**

HRQoL (both physical and mental) is a key factor for preventing maladjustment in chemotherapy patients. Social support and positive thinking coping style fosters fighting spirit and fatalism on health outcomes. Two potential lines of action seem promising: preventing maladaptive and promoting adaptive adjustments working on patient's mental health individually and involving significant others in supportive care, respectively.

## 1. Introduction

Considering the patient as an individual situated in a social context and dealing with the subjective experience of the illness in parallel with clinical data, the biopsychosocial model has prompted a paradigm shift in medicine, from disease-centered to patient-centered care [1]. Along with physiological or biochemical data, the patient's self-assessment of physical, social, and mental health, namely, the Health-Related Quality of Life (HRQoL), has become increasingly important in research and clinical practice. Different from the quality of life, which refers to the impact of various socioeconomic factors (e.g., wealth, education, pollution) on the welfare of every human being, HRQoL deals with the impact of a disease on clinical patients in terms of limitations of daily activities because of poor physical, mental, and social health. In this framework, oncology has paved the way for studies involving HRQoL, becoming a primary outcome for interventions aimed at improving the management of clinical symptoms and side effects of the treatment [2–4]. In the present study, we propose a heuristic model for the interrelations among biopsychosocial correlates of adjustment to cancer in which HRQoL has a crucial role in describing how patients meet the physical and emotional challenges of cancer during chemotherapy. Before presenting empirical data in support of specific hypotheses, we review the literature that has inspired the study.

From the moment of diagnosis, cancer patients face a new life situation and an uncertain future that is a source of apprehension and existential suffering [5]. In subsequent stages, the physical and psychological symptoms increasingly interfere with daily living, limiting the social roles within the family and community [6]. Therefore, patients must adjust to the stress of cancer and its treatment in an ongoing process that follows the course of the disease and proceeds with changes in HRQoL [7]. Anxious preoccupation and helplessness-hopelessness reflect a maladaptive adjustment pattern. In normal adjustment, instead, the patient learns how to manage cancer-related issues and how to cope with emotional distress effectively. Adaptive adjustment is often described using terms like “fighting spirit” (e.g., taking up actions to overcome the disease) and “fatalism” (e.g., accepting the situation without resignation) [8–10]. Cognitive avoidance (e.g., escaping cancer-related thoughts) is another form of adjustment, whose adaptive or maladaptive role is not clear [11].

Which factors do predict positive adjustment and better quality of life? Several studies investigated age and gender. In general, older patients have fewer adjustment problems than younger ones [12–16]. Age differences in HRQoL have been studied with mixed results. For instance, younger patients report higher scores on physical functioning but lower scores on social functioning [17]. Regarding gender, women with cancer experience more distress, anxiety, and depression than men [12, 16, 18–20] as well as more impairments in all HRQoL domains [17]. A biomedical variable that is frequently associated with the adjustment to cancer is the stage of the disease. It is logical to expect that more advanced patients experience higher levels of anxiety, depression, and hopelessness [21–25]. Surprisingly, however, other studies failed to support this relationship [3, 26–29]. With few exceptions [30], an advanced stage is associated with worse quality of life in different types of cancer [31–33].

Are quality of life and adjustment to cancer interrelated? Previous research has provided an affirmative answer [34–36]. Although the studies viewed HRQoL to be a consequence of adjustment, a reciprocal relationship is likely. First, better HRQoL itself denotes more positive adjustment to chronic disease [22, 37]. Second, from a biopsychosocial perspective, not only specific types of adjustment can influence HRQoL, but also how patients perceive their health status can influence the way in which they adjust to cancer [9, 38, 39]. Third, because HRQoL reflects the functional effects of clinical symptoms and side effects of the treatment, it is a direct indicator of the burden of the disease that patients are called upon to adapt to [40–42]. Taken together, the perceived severity of the physical symptoms can explain how the course of the disease influence the mental adjustment to cancer, with several social and personality factors involved in this process, the two most important being social support and optimistic coping styles [40].

By increasing the motivation to take care of oneself, providing tangible help, or addressing fears and concerns about the disease, family, friends, and significant others make an essential contribution to the supportive care of patients. Not surprisingly perceived social support is associated with better mental health and HRQoL [43, 44]. Regarding adjustment, Yağmur & Duman [45] showed that higher perceptions of social support were related to greater fighting spirit and lesser helplessness-hopelessness and fatalism. Likewise, Kawa [46] reported significant correlations between fighting spirit and the amount of support received from the social network, while others showed that higher levels of social support were associated with greater resilience and lower risk of anxiety and depression [47, 48].

Although adjustment to cancer refers to how patients cope with a cancer-related problem at specific points in time, ways of coping and their adaptational outcomes are separate concepts [49]. Indeed, coping styles are enduring dispositions that drive habitual appraisal and coping endeavors [50] and correlate with psychosocial outcomes in cancer patients [51, 52]. Positive thinking is one of such styles that has been extensively studied [28, 53]. For instance, maintaining a positive outlook during the treatment protected patients from maladjustment [54–56] and predicted better quality of life [57, 58].

**Figure 1:**
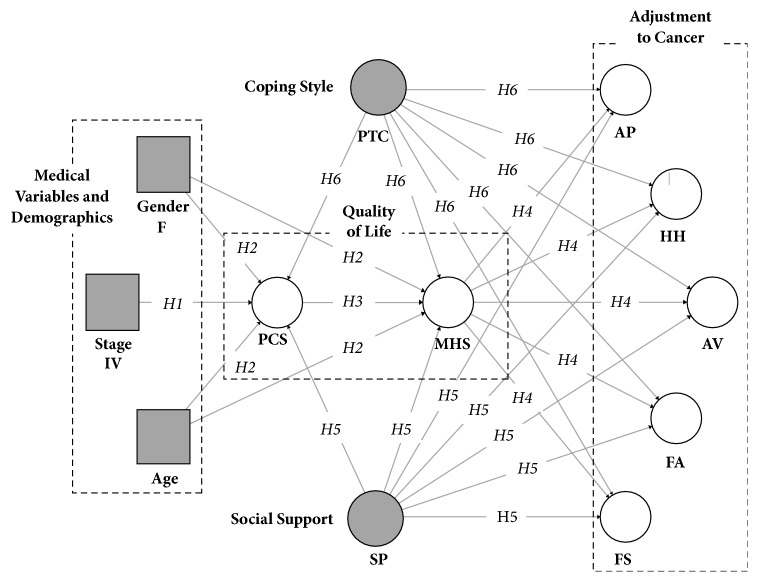
Theoretical model and hypotheses.* Legend*: Age = patient's age. Gender F = female versus male gender. Stage IV = Stage IV versus Stage I-III. PCS = Patient's Physical Functioning. MHS = Patient's Mental Health. AP = anxious preoccupation. AV = Cognitive Avoidance. FA = Fatalism. FS = Fighting Spirit. HH = Helplessness-Hopelessness. PTC = Positive Thinking Coping Style. SP = Social Provision.

Our model considers the interplay of illness-related and demographic variables, quality of life outcomes, individual differences in positive thinking, and social support provided to the patient. Each class of variables has been studied, often individually, in relation to adjustment to cancer, in some cases with mixed results. A lesser number of studies investigated the joint effects of these variables. As shown in Figure 1, we consider age, gender, and advanced cancer stage as the most distal antecedent of adaptational outcomes. Instead, we posit that demographics and medical variables are more proximal to the functional consequences of symptoms and side effects of the therapy (H1-H2). Although physical and mental aspects of HRQoL are intertwined, we considered physical functioning to be upstream of mental functioning (H3). Because patients with a history of mental illness were not eligible for the present study, we assume that an altered mental status is likely due to poorer physical functioning, not the other way around. Quality of life has a central role in the model, with physical functioning and mental health associated with different types of adjustment indirectly and directly, respectively (H4). Last, we included in the model two additional influences on adjustment: social support and positive thinking. In keeping with the literature, we expect these variables to be related to better quality of life and positive adjustment to cancer (H5-H6).

## 2. Materials and Methods

### 2.1. Participants

The data were collected as part of a larger study on the perceived quality of healthcare in oncology settings. One-hundred-sixty-two consecutive patients were recruited from two outpatient cancer treatment centers in the city of Rome, Italy. All participants were receiving either adjuvant chemotherapy (after primary treatment) or standard chemotherapy to reduce tumor size or to prevent metastatic cancer from spreading. Preliminary descriptive analyses obtained from one of the two centers have been reported elsewhere [59]. Inclusion criteria for the study were a performance status (ECOG) of 0 or 1, written comprehension of the Italian language, ability to fill in a paper and pencil questionnaire, and age over 18 years old. Exclusion criteria were refusal to cooperate and present or history of mental illness. Oncology doctors provided medical information about stage and type of cancer and checked the exclusion criteria during anamnesis. In particular, they asked the patients whether they ever had or have been told by a health professional to have a mental illness and which medications they are currently taking (subsequently coded as psychotropics or narcotics to be used as a proxy of present mental illness). The ethical review board of the hospital approved all aspects of the study. After we informed patients about the voluntary nature of participation and the right to withdraw from the study at any moment, we obtained their verbal consent before data collection. The refusal rate was around 5%.  Table 1 reports demographics and illness-related data.

### 2.2. Instruments and Variables

#### 2.2.1. Adjustment to Cancer

The Italian version of the Mini-Mental Adjustment to Cancer [60] includes 29 items tapping into three adaptive (cognitive avoidance, fatalism, and fighting spirit) and two maladaptive dimensions of adjustment to cancer (helplessness-hopelessness and anxious preoccupation). The questionnaire used a 4-point rating scale (1= definitely does not apply to me and 4= definitely applies to me). To test the model depicted in Figure 1, we defined five latent variables, each measured by composite scores obtained averaging two or three items that belong to specific forms of adjustment.

#### 2.2.2. Quality of Life

The perceived physical functioning and mental health of the patients were assessed using the Italian version of the 12-item Short-Form Health Survey [61]. The survey uses various response scales. In data analysis, we used two latent variables obtained from the six items that belong to the physical and mental domains, respectively.

#### 2.2.3. Social Support

The SPS-10 was derived from the Social Provision Scale [62], selecting 2 items for each of the following 5 aspects: emotional support, social integration, reassurance of value, tangible and material assistance, and orientation. The SPS-10 uses a four-point Likert-type scale (1 = strongly disagree, 2 = disagree, 3 = agree, and 4 = strongly agree) and provides a total score of perceived social support. We averaged the two items for each aspect of social support to obtain empirical indicators of Social Provision in the model.

#### 2.2.4. Positive Thinking Coping

Following Baumstrack et al. [53], Positive Thinking Coping was measured using composite scores of humor, planning, and positive reframing, included in the Brief-Cope Inventory. The items used a dispositional response format that aims to capture dispositional coping styles asking patients to reveal what they habitually do when they are distressed (e.g., for reasons other than their health status). The response scale was Likert-type (1 = I do not do this at all and 4 = I do this a lot).

#### 2.2.5. Demographics and Illness-Related Variables

Patient's age, gender, cancer type, and cancer stage were retrieved from the medical chart. Cancer stage was coded as early stage (I-III or nonmetastatic) and advanced stage (IV or metastatic).

### 2.3. Data Analysis

We performed a partial least squares structural equation modeling analysis (PLS-SEM) using Smart PLS 3 [63]. PLS-SEM is a prediction-oriented path analysis method recommended when the goal of the study is model building rather than theory testing [64]. PLS-SEM makes no assumptions regarding the underlying distribution of the variables, working well with nonnormal or highly skewed data [64]. The model tested has five exogenous variables, three of which (cancer stage, gender, and age) are manifest variables measured without error, and two of which (social support and positive thinking coping) are latent variables (Figure 1). Because some cancer types overlap with gender (e.g., prostate, breast), cancer type was omitted from the model. The model has seven endogenous latent variables representing patients perceived physical functioning, mental health, anxious preoccupation, helplessness-hopelessness, cognitive avoidance, fatalism, and fighting spirit (Figure 1).

Model evaluation comprises two stages: the assessment of the “measurement model”, dealing with the relationships between the empirical indicators and the latent variables, and the evaluation of the “structural model”, which represents the direct and indirect relationships between latent variables. Four quality criteria determine the adequacy of the measurement model. First, all indicators variables should load on the corresponding latent variables above 0.50 (indicator reliability). Second, the composite reliability (CR) of each latent variable should be at least above 0.60, or preferably above 0.70 (construct reliability). Third, the Average Variance Extracted (AVE), measuring the proportion of variance in the indicators that is accounted for by the corresponding latent variable, should be 0.50 or higher (convergent validity). Last, the square roots of the AVE for each latent variable should be larger than the estimated correlations of that latent variable with other variables in the model (discriminant validity).

The evaluation of the structural model is based on how well the model predicted the endogenous variables. First, we examined the determination coefficients (R^2^) for the endogenous latent variables. According to Hair et al. [64], R^2^ values of 0.75, 0.50, and 0.25 represent high, moderate, and low thresholds, respectively. The predictive accuracy of the model is also evaluated in terms of cross-validation (i.e., the ability of the model to predict omitted data not used for estimation). For this purpose, a Q^2^ cross-validation index is obtained for each endogenous variable using a blindfolding procedure [64]. Positive Q^2^ values indicate that the model has a predictive relevance. The higher the Q^2^, the higher the predictive accuracy of the model. The significance of the direct path coefficients is tested using nonparametric confidence intervals obtained from 5000 bootstrap resampling iterations [65]. Besides evaluating the significance of the path coefficients, it is advised to assess their effect size. This assessment is typically done by computing the f^2^, which is the change in R^2^ in an endogenous variable when a specific path is omitted from the model. Following Hair et al. [64], 0.02, 0.15, and 0.35 represent small, medium, and large effect sizes, respectively.

## 3. Results and Discussion

### 3.1. Measurement Model

Table 2 reported the reliability statistics needed to assess the quality of the measurement model. The composite reliability indexes were above the recommended threshold of 0.70, ranging from 0.79 to 0.90 for all the latent variables in the model. Because stage, age, and gender are manifest variables measured without error, their composite reliability is by definition equal to 1.00 (omitted from Table 2). The AVE index for the latent variables was higher than the recommended standard of 0.50 except for positive thinking coping whose value (i.e., 0.49) was just 0.01 units below the standard. The square roots of the AVE (in the diagonal of Table 2) were higher than the correlations of the latent variables with other latent variables in the model (Table 2), thus meeting with the criterion for discriminant validity.

All indicators loaded on the respective latent variable much above 0.50, attaining the standard for indicator reliability. In few cases, the indicator variables cross-loaded another latent variable above 0.50. In no case, the indicator variables loaded more on the other latent variables than they did on the latent variable they were supposed to measure. The full factor loading matrix is reported in supplementary materials (available here).

Taken together, the analyses of the measurement model have shown that the composite and indicator reliability, as well as the convergent and discriminant validity of the constructs, were acceptable.

### 3.2. Structural Model

**Figure 2:**
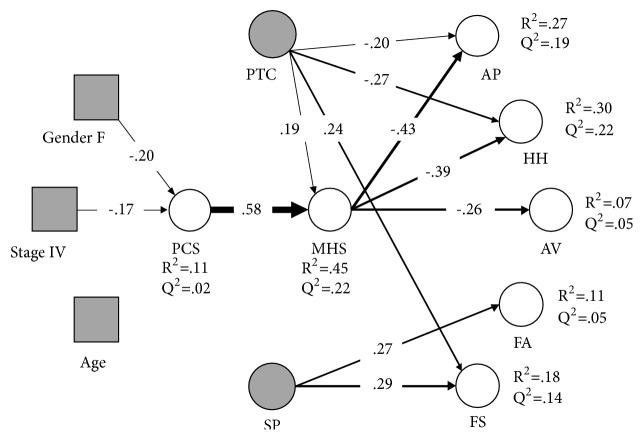
The estimated structural model.* Note*: all paths were significant at p <.05. Line thickness is proportional to the size of the standardized regression coefficients. Nonsignificant regression paths have been omitted.* Legend*: Age = patient's age. Gender F = female versus male gender. Stage IV = Stage IV versus Stage I-III. PCS = Patient's Physical Functioning. MHS = Patient's Mental Health. AP = anxious preoccupation. AV = Cognitive Avoidance. FA = Fatalism. FS = Fighting Spirit. HH = Helplessness-Hopelessness. PTC = Positive Thinking Coping Style. SP = Social Provision.

Figure 2 showed the estimated structural model, including the R^2^ and Q^2^ statistics for the endogenous variables. The model explained 27% and 30% of the variance in anxious preoccupation and helplessness-hopelessness, 18% and 11% of the variance in fighting spirit and fatalism, and 7% of the variance in cognitive avoidance, respectively. According to Hair et al. [64], the effect sizes were above the small-medium for anxious preoccupation and helplessness-hopelessness, slightly below the small size threshold for fatalism and fighting spirit and much below the threshold for cognitive avoidance. The effect size for mental health approached the moderate level, while that for the physical functioning was rather small. The Q^2^ values were all positive, supporting the predictive quality of the model regarding cross-validation capacity, especially for anxious preoccupation, helplessness-hopelessness, and mental health.

Table 3 reported the tests of model coefficients. The cancer stage was directly associated with the perceived physical functioning, so that stage IV cancer patients reported poorer physical functioning than early stage patients. Age was neither associated with physical functioning nor with patient mental health. Female gender was negatively associated with physical functioning so that females reported more complaints regarding mobility, pain, and limitations of daily activities due to a health condition. The most robust relationship in the model was the link between physical functioning and mental health. In turn, mental health was inversely related to anxious preoccupation, helplessness-hopelessness, and cognitive avoidance (in order of relative importance). Unexpectedly, better mental health was not associated with greater fighting spirit, while the path coefficient of mental health with fatalism was only marginally significant. The tests of model's coefficients showed that higher social support provided to the patients was associated with more fighting spirit and more fatalism. Positive thinking coping was positively associated with fighting spirit and negatively with anxious preoccupation and helplessness-hopelessness. Positive thinking coping was also linked with mental health.

The effect sizes of each path were appraised using the f^2^. As one can see from Table 3, the relationship between physical functioning and mental health was of substantial importance, while the relationships of mental health with anxious preoccupation and helplessness-hopelessness were of medium-large effect sizes. The relationships of social support with fighting spirit and fatalism were both in the small-medium range. Regarding positive thinking coping, the relationships of this variable with the latent variables representing the adjustment to cancer were also small-medium. The effect of disease stage and gender on the physical functioning was barely small.

The PLS model allowed us to explore possible indirect relationships of the stage, age, and gender with adjustment to cancer. As one can see from Table 4, both stage and gender were associated with anxious preoccupation and helplessness-hopelessness through worse quality of physical functioning and mental health. Although the standardized indirect effects were small (i.e., about 0.05 standard deviations in adjustment were explained by stage and gender), the finding suggested that gender and stage might be related to negative types of adjustment because of worse physical functioning and subsequently through worse mental health.

## 4. Conclusion

Which factors are associated with adjustment to cancer in chemotherapy patients? Previous research has considered illness-related variables and demographics, quality of life, personality, and social factors to affect psychosocial outcomes in cancer patients [40]. Central to our study was the assumption that disease progression increasingly compromises the patient's physical functioning, leading to worsening mental health and reducing the ability to manage cancer-related issues and emotional distress effectively. In keeping with the literature, we found a negative association between advanced stage and physical functioning [31–33], a strong positive link between physical functioning and mental health [9, 38, 39], and significant relations between mental health and adaptational outcomes [34–36].

In a biopsychosocial perspective, the medical characteristics of the disease and quality of life are intertwined [1]. Accordingly, our data showed that cancer stage was the most distal antecedent of adjustment, influencing physical functioning directly and both mental health and adjustment indirectly. Because present or history of mental illness was an exclusion criterion for our study, we interpreted the strong association between physical functioning and mental health considering that a worse mental status could be a consequence of a compromised physical status. This interpretation is consistent with previous studies in which the patients' health appraisals shaped how they adjusted to cancer [9, 38, 39].

Gender differences are widely acknowledged in the biological–biomedical aspects of clinical care. Previous research agrees that women with cancer tend to report more depression, pain, and disability than men [12, 16–20]. Likewise, in our study, women adjusted to cancer with more anxious preoccupation and helplessness-hopelessness than men. Yet, our model showed that female gender was no longer associated with the two negative aspects of adjustment when controlling for HRQoL. Expanding on previous research, this finding suggests that women with cancer tend to be more depressed by and more concerned about their condition than men to the extent that they experience deterioration in physical functioning and the associated psychological and social consequences.

Age differences are also believed significant predictors of adjustment to cancer. Previous studies have shown greater adjustment problems for the youngest patients [12–16]. Unexpectedly, however, we did not find any significant effect of age on adaptational outcomes nor associations with HRQoL. In default of any better explanation, we interpreted this unexpected finding considering that one cannot take for granted that patients differing in age also differ in age-related psychological strengths and weaknesses that might account for positive or negative types of adjustment. Alternatively, the lack of correlation between age and adjustment to cancer in our sample might also reflect the fact the chemotherapy treatment could be a difficult period for patients of all ages, mitigating the expected correlations between age and the variables in the model.

In line with our hypothesis, mental health was associated with anxious preoccupation, helplessness-hopelessness, and avoidance. Better mental health can protect patients from increased feelings of loss, despair, and discouragement, and from pervasive, or uncontrollable, preoccupations, worries, and fears. This finding underscores the need for chemotherapy patients to maintain positive and repair negative moods as a protective factor against maladjustment. Research has long debated on whether cognitive avoidance is adaptive or maladaptive for cancer patients [11]. In keeping with the literature [49, 60], we found that avoiding thinking about the disease and its implications was related to both maladaptive and adaptive types of adjustment. The relationships with the fighting spirit and fatalism suggest that cognitive avoidance reflected a conscious escape, actively sought by chemotherapy patients, to cope with the burden of the disease and its treatment. Paradoxically, however, cognitive avoidance is a coping strategy known to relieve anxiety in the short-term, turning out to be ineffective in the long run, a view consistent with the unique associations of cognitive avoidance with poor mental health and anxious preoccupation in the present study.

Our model showed that the interplay of dispositional and social factors accounted for an adequate adjustment. To cope with the broad range of physical and emotional challenges imposed by the disease, chemotherapy patients needed help from other people, often living together. Significant others and informal social support networks are essential for the patient's psychological adaptation to physical illness, fostering fatalistic attitudes and fighting spirit [43–48].

The word fatalism comes from the Latin term “fatum” (literally, destiny) referring to passive acceptance of events. From this perspective, a fatalistic attitude becomes maladaptive if passive surrender prevails for an extended period engendering hopelessness and despair [35]. However, fatalism can also be considered an adaptive response that promotes, or preserves, the well-being of the patient, especially in specific periods (e.g., the diagnosis) or limited time (e.g., waiting for test results after a therapy cycle) [9, 10]. In fact, a fatalistic attitude might reflect a genuine acceptance of the existing situation accompanied by a positive reframing of the stressful experience that favors an adaptive adjustment [8, 49].

Our data showed that for chemotherapy patients to develop, or preserve, a strong determination to overcome the disease, the supportive capacities of their network and a positive thinking disposition are essential resources that, if strengthened, might help them to experience more control over cancer-related problems and maintain faith in treatment. Although some scholars have dismissed the idea that fighting spirit is associated with “hard” medical outcomes [66], there is also evidence that greater fighting spirit, before and during chemotherapy, predicts disease-free and overall survival [67]. Doubtless, the fighting spirit can reduce patients distress and helplessness during the disease [8, 9, 35]. Notwithstanding this, some eminent organizations (e.g., American Cancer Society) expressed serious concerns regarding encouraging the fighting spirit and instilling optimism in cancer patients. In fact, exerting generic pressure on patients to adopt culturally prescribed coping strategies might be even counterproductive, generating feelings of inadequacy or guilt to those who fail to comply [68, 69].

In keeping with the literature [55–58], our model showed that patients who* habitually* reframe any stressful situations maintaining a positive outlook, were more protected from negative adjustment, had significantly better mental health, and were “fighting” the disease with the usual energy and positive attitude. Because coping styles are enduring dispositions of the person [50], it is difficult to think that personality factors can be a resource available to everyone to cope with the stress of the disease. However, because psychological outlooks are malleable [54], patients who lack dispositional optimism could be targeted for psychological interventions to prevent pessimism from turning into hopelessness and despair.

This study has some noteworthy limitations. First, the sample size was relatively small. Although the number of patients was adequate for performing PLS-SEM analyses and testing both direct and indirect relationships, it precluded us from better stratifying the stage of the disease (e.g., equal number of cases in each of the four stages) or considering the primary tumor site as a multicategorical predictor that could influence one's adjustment to cancer. Second, the cross-sectional design impedes the inference of causal relationships. All interpretations of directional effects are based on previous theory and research. Even though the biopsychosocial model assumes reciprocal relationships among biological, psychological, and social factors, future research should rely on longitudinal design or intervention studies, which might help to disentangle how social support, coping styles, and changes in HRQoL affect adjustment to cancer at specific points in the course of the disease. Third, the findings of the present study cannot be generalized beyond out-patients receiving chemotherapy. Other groups of cancer patients should be included to generalize our findings, such as those who have just been diagnosed or those on their way to palliative care. Fourth, we assessed a present or a history of mental illness using self-report and proxy measures of psychopathology instead of relying on a structured psychiatric interview. Despite the fact that using psychotropic drugs is commonly regarded as an indicator of mental illness [70], still it is entirely possible that some participants had undiagnosed mental disorders that could have been screened only using a diagnostic interview. Last, the timing of evaluation did not consider the course and the chemotherapy and different levels of side effects interfering with the health status. Although this might represent an uncontrolled source of variance, we believe the HRQoL measures included in the present study reflect the subjective experience of both positive and negative effects of the treatment on patients' day-to-day live [2–4].

Notwithstanding these limitations, the results of the study, albeit preliminary, showed that HRQoL (both physical and mental) is a key factor in our model along with social support and positive thinking coping. We hope our study will inspire further research in this area and facilitate clinicians to rethink interventions aimed to improve adjustment to cancer in chemotherapy patients. Two lines of action seem promising: preventing maladaptive and promoting adaptive adjustments working on mental health and social support, respectively. To attain the first goal, clinical interventions should target early signs of mental problems before they become chronic or such severe to limit daily activities. Because the failure of the adaptation process seems unrelated to the scarcity of resources in the social network, or limits in the contexts of belonging, a preventive intervention should target internal processes, in priority, improving one's ability to regulate negative emotions. To promote an adaptive adjustment, enhancing the fighting spirit, the focus of clinical interventions should be on evaluation and activation of the social resources of the patient, especially for those who have a narrow or qualitatively poor social support network.

Family, friends, and significant others* must *become a resource to be actively used by the patient or reconstructed if lacking. In response to concerns regarding the generalized prescription of fighting spirit, the social network, if enriched, could naturally provide the patient with enough reasons to engage in his/her struggle with the disease or favoring optimistic appraisals in the moments of greatest distress in the journey of the illness. We hope that the assessment of patients' resources, regarding mental health and social support, will be included in standard protocols just after communicating the diagnosis. This would help planning early interventions on cancer patients, especially the most vulnerable, who need additional psychological care to improve their ability to deal with a complex and painful event such as the experience of cancer.

## Figures and Tables

**Table 1:** Demographics and illness-related data.

Patient Characteristics			
Variables		N	%
1. Age (M±SD)	(58.97±13.28)		
2. Gender	Female	89	55.0
	Male	73	45.0
3. Primary Tumor Site	Digestive System/Abdomen	60	37.0
	Female Genital Apparatus	22	13.6
	Breast	25	15.4
	Respiratory System/Thorax	27	16.7
	Urinary apparatus	12	7.4
	Male genital apparatus	8	4.9
	Other/Unspecified	8	4.9
4. Disease Stage	I-III	61	37.7
	IV	101	62.4

*N*=162.

**Table 2:** Reliability and validity of the latent variables.

Latent Variable	AVE	CR	1.		2.		3.		4.		5.		6.		7.		8.		9.	
1. Anxious Preoccupation (AP)	.85	.92	**.92**																	
2. Avoidance (AV)	.76	.90	.43	*∗∗∗*	**.87**															
3. Fatalism (FA)	.88	.94	.09		.24	*∗∗*	**.93**													
4. Fighting Spirit (FS)	.65	.79	.03		.22	*∗∗*	.35	*∗∗∗*	**.81**											
5. Helplessness-Hopelessness (HH)	.80	.92	.56	*∗∗∗*	.16	*∗*	.00		-.14		**.89**									
6. Mental Health (MH)	.57	.80	-.49	*∗∗∗*	-.24	*∗∗*	-.06		.09		-.48	*∗∗∗*	**.75**							
7. Physical Health (PH)	.55	.88	-.27	*∗∗∗*	-.03		.04		.04		-.28	*∗∗∗*	.63	*∗∗∗*	**.74**					
8. Positive Thinking Coping (PTC)	.49	.85	-.32	*∗∗∗*	-.05		.16	*∗*	.33	*∗∗∗*	-.39	*∗∗∗*	.30	*∗∗∗*	.16		**.70**			
9. Social Provision (SP)	.59	.88	-.14		.07		.28	*∗∗∗*	.37	*∗∗∗*	-.19	*∗*	.21	*∗∗*	.14		.35	*∗∗∗*	**.77**	

*Note*: *∗∗∗* p<.001; *∗∗* p<.01; *∗* p< .05. Bold coefficients in the diagonal are the squared roots of AVE. Coefficients below the diagonal are correlations among the latent variables in the model.

*Legend*: AVE = Average Variance Extracted; CR = composite reliability.

**Table 3:** Tests of model coefficients.

Path	Beta	t-value	p	f^2^	Effect size
Age -> MHS	.06	.90	.370	.01	Null
Age -> PCS	-.06	.76	.450	.00	Null
Gender F -> MHS	-.07	1.20	.230	.01	Null
*Gender F -> PCS*	*-.20*	*2.51*	*.010*	*.04*	*Small medium*
*Stage IV -> PCS*	*-.17*	*2.27*	*.020*	*.03*	*Small medium*
*PCS -> MHS*	*.58*	*11.34*	*.001*	*.57*	*Large*
*MHS -> AP*	*-.43*	*7.42*	*.001*	*.23*	*Medium Large*
*MHS -> AV*	*-.26*	*3.49*	*.001*	*.06*	*Small medium*
MHS -> FA	-.16	1.91	.060	.02	Small
MHS -> FS	-.04	.60	.550	.00	Null
*MHS -> HH*	*-.39*	*5.22*	*.000*	*.20*	*Medium Large*
*PTC -> AP*	*-.20*	*2.51*	*.010*	*.05*	*Small medium*
PTC -> AV	-.02	.19	.850	.00	Null
PTC -> FA	.11	1.39	.170	.01	Null
*PTC -> FS*	*.24*	*2.84*	*.001*	*.06*	*Small medium*
*PTC -> HH*	*-.27*	*3.59*	*.001*	*.08*	*Small medium*
*PTC -> MHS*	*.19*	*2.79*	*.010*	*.05*	*Small medium*
PTC -> PCS	.10	1.19	.230	.01	Null
SP -> AP	.02	.27	.790	.00	Null
SP -> AV	.13	1.70	.090	.02	Small
*SP -> FA*	*.27*	*3.69*	*.001*	*.07*	*Small medium*
*SP -> FS*	*.29*	*4.01*	*.001*	*.09*	*Small medium*
SP -> HH	-.02	.20	.840	.00	Null
SP -> MHS	.07	1.21	.230	.01	Null
SP -> PCS	.10	1.18	.240	.01	Null

*Note*: according to Cohen's guidelines, f^2^ ≥ 0.02, f^2^ ≥ 0.15, and f^2^ ≥ 0.35 represent small, medium, and large effect sizes.

*Legend*: Age = patient's age. Gender F = female vs. male gender. Stage IV = Stage IV vs. Stage I-III. PCS = Patient's Physical Functioning. MHS = Patient's. AP = anxious preoccupation. AV = Cognitive Avoidance. FA = Fatalism. FS = Fighting Spirit. HH = Helplessness-Hopelessness. PTC = Positive Thinking Coping Style. SP = Social Provision. Italicized text highlights statistically significant paths.

**Table 4:** Bootstrap tests of indirect effects identified in the structural model.

Indirect Effect	Point Estimate		Bias	LLCI	ULCI
	Sample	Bootstrap			
Age -> PCS -> MHS -> AP	.03	.03	.00	-.01	.07
Age -> PCS -> MHS -> AV	-.02	-.01	.01	-.07	.02
Age -> PCS -> MHS -> FA	-.01	.00	.01	-.05	.01
Age -> PCS -> MHS -> FS	.00	.00	.00	-.03	.00
Age -> PCS -> MHS -> HH	.02	.02	.00	-.01	.07

*Gender F -> PCS -> MHS -> AP*	*.05*	*.05*	*.00*	*.01*	*.10*
Gender F -> PCS -> MHS -> AV	-.03	-.01	.02	-.09	.03
Gender F -> PCS -> MHS -> FA	-.02	.00	.02	-.07	.02
Gender F -> PCS -> MHS -> FS	.00	.00	.00	-.03	.01
*Gender F -> PCS -> MHS -> HH*	*.05*	*.05*	*.00*	*.01*	*.09*

*Stage IV -> PCS -> MHS -> AP*	*.04*	*.04*	*.00*	*.01*	*.09*
Stage IV -> PCS -> MHS -> AV	-.03	-.01	.02	-.07	.03
Stage IV -> PCS -> MHS -> FA	-.02	.00	.01	-.07	.01
Stage IV -> PCS -> MHS -> FS	.00	.00	.00	-.03	.01
*Stage IV -> PCS -> MHS -> HH*	*.04*	*.04*	*.00*	*.01*	*.09*

*Legend*: LLCI = 99% Lower Limit Confidence Interval. ULCI = 99% Upper Limit Confidence Interval. Bias = difference between sample and bootstrap and sample estimates of indirect effects. Age = patient's age. Gender F = female vs. male gender. Stage IV = Stage IV vs. Stage I-III. PCS = Patient's Physical Functioning. MHS = Patient's. AP = anxious preoccupation. AV = Cognitive Avoidance. FA = Fatalism. FS = Fighting Spirit. HH = Helplessness-Hopelessness. Italicized text highlights statistically significant paths.

## Data Availability

The dataset generated and analyzed during the current study is available from the corresponding author upon reasonable request.

## Supplementary Materials

Supplementary Materials Supplementary Table: factor loadings (bolded) and cross-loadings.
