# Supplementary material for: Biopsychosocial Correlates of Adjustment to Cancer during Chemotherapy: The Key Role of Health-Related Quality of Life
Source: ScientificWorldJournal. 2019 Mar 10;2019:9750940. doi: 10.1155/2019/9750940 (PMC6431483; doi:10.1155/2019/9750940)
Supplement: Supplementary Materials — Supplementary Table: factor loadings (bolded) and cross-loadings. [file 9750940.f1.docx]

**Supplementary Table.** Factor loadings (bolded) and cross-loadings.

| **Latent Variable** |  | **AV** | **AP** | **FS** | **FA** | **HH** | **PTC** | **MHS** | **PCS** | **SP** | **STA** | **AGE** | **GEF** |
| --- | --- | --- | --- | --- | --- | --- | --- | --- | --- | --- | --- | --- | --- |
|  | Indicator |  |  |  |  |  |  |  |  |  |  |  |  |
| **Avoidance (AV)** | AV1 | **.93** | .42 | .30 | .22 | .14 | .02 | -.23 | -.04 | .08 | .05 | .20 | .16 |
|  | AV2 | **.91** | .36 | .09 | .23 | .15 | -.12 | -.21 | -.03 | .05 | .08 | .16 | .16 |
| **Anxious Preoccupation (AP)** | AP1 | .33 | **.88** | -.01 | .05 | .53 | -.35 | -.41 | -.24 | -.15 | .04 | -.02 | .17 |
|  | AP2 | .40 | **.85** | .12 | .10 | .48 | -.29 | -.39 | -.19 | -.15 | .04 | .00 | .16 |
|  | AP3 | .39 | **.88** | -.04 | .08 | .44 | -.21 | -.47 | -.26 | -.06 | .12 | .11 | .13 |
| **Fighting Spirit (FS)** | FS1 | .28 | .05 | **.94** | .40 | -.14 | .32 | .09 | .05 | .34 | .00 | -.01 | .11 |
|  | FS2 | .13 | .00 | **.94** | .25 | -.13 | .29 | .09 | .03 | .35 | .02 | -.05 | .03 |
| **Fatalism (FA)** | FA1 | .12 | -.01 | .22 | **.79** | .00 | .11 | -.07 | .00 | .21 | .09 | .00 | .15 |
|  | FA2 | .27 | .14 | .34 | **.83** | .00 | .15 | -.04 | .07 | .24 | .01 | -.02 | .10 |
| **Helplessness-hopelessness (HH)** | HH1 | .20 | .53 | -.11 | .06 | **.90** | -.35 | -.47 | -.21 | -.08 | .10 | -.01 | .10 |
|  | HH2 | .11 | .49 | -.14 | -.02 | **.89** | -.38 | -.36 | -.31 | -.26 | .17 | -.03 | .06 |
|  | HH2 | .10 | .47 | -.13 | -.05 | **.89** | -.32 | -.44 | -.24 | -.19 | .15 | -.09 | .02 |
| **Positive Thinking Coping (PTC)** | PTC1 | -.11 | -.32 | .29 | .17 | -.37 | **.84** | .29 | .17 | .37 | -.14 | -.04 | -.07 |
|  | PTC2 | -.03 | -.15 | .12 | .10 | -.21 | **.69** | .19 | .10 | .20 | -.18 | -.02 | .01 |
|  | PTC3 | .05 | -.22 | .29 | .08 | -.27 | **.72** | .18 | .08 | .17 | -.06 | .03 | .13 |
| **Mental Health (MH)** | MH1 | -.23 | -.41 | .10 | -.04 | -.40 | .25 | **.78** | .36 | .16 | -.06 | .03 | -.03 |
|  | MH2 | -.28 | -.54 | .14 | -.09 | -.49 | .33 | **.81** | .40 | .19 | -.04 | -.04 | -.21 |
|  | MH3 | -.17 | -.23 | -.02 | -.09 | -.26 | .20 | **.67** | .46 | .03 | -.05 | -.05 | -.14 |
|  | MH4 | -.14 | -.23 | .07 | -.09 | -.27 | .17 | **.67** | .43 | .16 | -.02 | -.02 | -.10 |
|  | MH5 | -.17 | -.39 | .10 | .05 | -.39 | .19 | **.76** | .57 | .25 | -.03 | -.01 | -.14 |
|  | MH6 | -.03 | -.28 | -.01 | -.04 | -.24 | .17 | **.72** | .61 | .12 | -.17 | -.03 | -.12 |
| **Physical Health (PH)** | PH1 | .04 | -.25 | .05 | .03 | -.22 | .13 | .45 | **.71** | .14 | -.03 | -.02 | -.16 |
|  | PH2 | -.17 | -.33 | .09 | -.12 | -.25 | .14 | .45 | **.58** | .00 | -.19 | -.23 | -.10 |
|  | PH3 | -.01 | -.16 | -.05 | .02 | -.16 | .08 | .45 | **.79** | .06 | -.29 | -.12 | -.11 |
|  | PH4 | -.07 | -.18 | .02 | .08 | -.19 | .12 | .42 | **.77** | .14 | -.21 | -.05 | -.18 |
|  | PH5 | .10 | -.07 | .03 | .10 | -.18 | .11 | .46 | **.72** | .12 | -.02 | -.08 | -.19 |
|  | PH6 | -.01 | -.11 | .02 | .08 | -.17 | .08 | .40 | **.61** | .11 | -.02 | .06 | -.05 |
| **Social Provision (SP)** | SP1 | -.03 | -.09 | .25 | .15 | -.12 | .26 | .14 | .04 | **.80** | .00 | -.10 | .01 |
|  | SP2 | .04 | -.05 | .27 | .29 | -.08 | .23 | .14 | .07 | **.80** | .07 | -.06 | -.04 |
|  | SP3 | .14 | -.13 | .32 | .23 | -.22 | .27 | .17 | .12 | **.71** | .00 | -.01 | .05 |
|  | SP4 | .08 | -.10 | .30 | .19 | -.14 | .23 | .21 | .19 | **.77** | -.01 | -.10 | -.01 |
|  | SP5 | .01 | -.16 | .23 | .21 | -.16 | .34 | .15 | .07 | **.77** | .08 | -.15 | .01 |
| **Stage** | Stage IV | .07 | .08 | .01 | .06 | .15 | -.16 | -.08 | -.19 | .03 | **1.00** | .11 | -.04 |
| **Age** | Age | .20 | .03 | -.03 | -.02 | -.05 | -.02 | -.03 | -.11 | -.10 | .11 | **1.00** | .11 |
| **Gender** | Gender F | .17 | .18 | .08 | .15 | .07 | .01 | -.17 | -.19 | .00 | -.04 | .11 | **1.00** |
|  |  |  |  |  |  |  |  |  |  |  |  |  |  |
|  |  |  |  |  |  |  |  |  |  |  |  |  |  |

**Legend:** AV1 = Sum of Mini-MAC items 26 and 11. AV2 = Sum of Mini-MAC items 27 and 17. AP1 = Sum of Mini-MAC items 29, 22, and 5. AP2 = Sum of Mini-MAC items 13 and 5. AP3 = Sum of Mini-MAC items 28 and 9. FS1 = Sum of Mini-MAC items 2 and 10. FS2 = Sum of Mini-MAC items 23 and 24. FA1 = sum of Mini-MAC items 19 and 3. FA2 = sum of Mini-MAC items 1 and 8. HH1 = sum of Mini-MAC items 4, 12, and 16. HH2 = sum of Mini-MAC items 6, 14, and 20. HH3 = sum of Mini-MAC items 7, 15, and 21. PTC 1 = sum of Brief Cope Items 20 and 24. PTC 2 = sum of Brief Cope Items 18 and 28. PTC 1 = sum of Brief Cope Items 12 and 17. MH1 = SF-12 mental health item 3. MH2 = SF-12 mental health item 4. MH3 = SF-12 Role Emotional item 2. MH4 = role emotional item 3. MH5 = SF-12 social functioning item 2. MH6 = SF-12 vitality item 2. PH1 = SF-12 body pain item 2. PH2 = SF-12 general health item 1. PH3 = SF-12 physical functioning item 2. PH4 = physical functioning item 4. PH5 = SF-12 role physical item 2. PH6 = SF-12 role physical item 3. SP1 = sum of Social Provision Scale item 8 and 5. SP2 = sum of Social Provision Scale item 13 and 20. SP3 = sum of Social Provision Scale item 11 and 17. SP4 = sum of Social Provision Scale item 1 and 23. SP4 = sum of Social Provision Scale item 12 and 16.
